# The alveolar macrophage toponome of female SP-A knockout mice differs from that of males before and after SP-A1 rescue

**DOI:** 10.1038/s41598-022-08114-2

**Published:** 2022-03-23

**Authors:** David S. Phelps, Vernon M. Chinchilli, Lili Yang, Debra Shearer, Judith Weisz, Xuesheng Zhang, Joanna Floros

**Affiliations:** 1 Penn State Center for Host Defense, Inflammation, and Lung Disease (CHILD) Research and Departments of Pediatrics, The Pennsylvania State University College of Medicine, Hershey, PA 17033 USA; 2 Public Health Sciences, The Pennsylvania State University College of Medicine, Hershey, PA 17033 USA; 3 Obstetrics and Gynecology, The Pennsylvania State University College of Medicine, Hershey, PA 17033 USA

**Keywords:** Biological techniques, Cell biology, Immunology, Biomarkers

## Abstract

Using the Toponome Imaging System (TIS), a serial immunostainer, we studied the patterns of expression of multiple markers in alveolar macrophages (AM) from female mice lacking surfactant protein A (SP-A knockouts; KO) after “rescue” with exogenous SP-A1. We also used a 7-marker subset to compare with AM from males. AM were harvested 18 h after intrapharyngeal SP-A1 or vehicle, attached to slides, and subjected to serial immunostaining for 12 markers. Expression of the markers in each pixel of the image was analyzed both in the whole image and in individual selected cells. The marker combination in each pixel is referred to as a combinatorial molecular phenotype (CMP). A subset of antibodies was used to compare AM from male mice to the females. We found: (a) extensive AM heterogeneity in females by CMP analysis and by clustering analysis of CMPs in single cells; (b) AM from female KO mice respond to exogenous SP-A1 by increasing CMP phenotypic diversity and perhaps enhancing their potential innate immune capabilities; and (c) comparison of male and female AM responses to SP-A1 revealed that males respond more vigorously than females and clustering analysis was more effective in distinguishing males from females rather than treated from control.

## Introduction

Surfactant protein A (SP-A) is the major protein component of pulmonary surfactant. It has also been identified in a variety of other tissues, including the intestine^1^, the nasal epithelium^2^, and the female genital tract^3^, as well as other sites. SP-A has been classified as a collectin or calcium-dependent collagenous lectin based on the fact that it contains a collagenous domain and a Ca^+2^-dependent carbohydrate recognition domain that binds specific carbohydrate components of cells, microorganisms, and carbohydrate-containing macromolecules in the lung^4^.

SP-A, like other collectins (including, but not limited to the mannose-binding lectin, complement C1q, conglutinin, and SP-D) appears to be of particular importance in host defense^4–7^. SP-A binds various pathogens, allergens, and particulates, affecting their recognition by immune cells. SP-A also regulates the function of immune cells themselves. This potentially enhances their ability to respond effectively to exposure to various substances, as well as their ability to mediate other inflammatory and repair processes in the lung. Humans, unlike rodents, have two genes for SP-A (*SFTPA1* and *SFTPA2*) encoding two closely related, but different proteins (SP-A1 and SP-A2)^8–10^. These are differentially regulated^11^ and there is abundant evidence that there are differences in the function of these two proteins, although both clearly enhance host defense function^10,12^.

We have investigated the effects of SP-A on alveolar macrophages (AM) and macrophage-like cell lines in a number of studies. These studies showed that SP-A can regulate the production of proinflammatory cytokines^13–15^. This action is mediated by NF-κB^13^ following the interaction of SP-A with TLR2^16^. SP-A also has significant stimulatory effects on bacterial phagocytosis^4,5^, both directly by serving as an opsonin^17,18^, and indirectly by enhancing the activity of the AM^19–21^. SP-A also causes changes in the AM proteome^22–24^, the actin cytoskeleton^25^, the miRNome^26–28^, and the toponome, the spatial network of potentially interacting proteins within the cell^29,30^. All of these actions are likely to influence host defense function^4^. An important example of the benefit of SP-A is shown by the greatly reduced survival in experimental bacterial or viral pneumonia models of mice lacking SP-A (SP-A knockout; KO) versus wild type mice^31–34^. Confirmation of the importance of SP-A is provided by an enhancement of survival in KO mice given exogenous SP-A and in transgenic mice on the SP-A KO background that express either human SP-A1 or SP-A2^35^.

SP-A1 and SP-A2 differentially affect the function and/or regulation of AM, or mouse survival after infection, as cited above, but SP-A2 seems to perform functions related to innate immunity more efficiently^10^. Conversely, various functions related to surfactant function and metabolism are more effectively performed by SP-A1^36^, and for the extracellular structural form of surfactant, tubular myelin, both SP-A1 and SP-A2 are needed^37^. Most of these SP-A1 and SP-A2 functional differences^10^ have been investigated in studies with humanized transgenic mice that express either SP-A1 or SP-A2, in experiments where SP-A KO mice are treated with exogenous SP-A1 or SP-A2 or where AM are treated in vitro with SP-A1 or SP-A2.

A number of studies have shown that the response to SP-A is sexually dimorphic^4,10,25–28,35,38^ and that sex hormones play a role^39^. Several studies investigating sex differences in survival and various AM processes under different conditions have concluded that females are more immunocompetent than males^24,26,28,35,38,40–42^. With this trend in mind, we wished to study CMP differences in the AM response to SP-A in males and females in an attempt to gain some insight into the complexity of AM sex-dependent responses that may underlie sex differences in lung innate immune function.

To investigate this, we employed the toponome imaging system (TIS). TIS is a microscopic system employing serial immunostaining that allows one to localize and compare multiple proteins in close proximity to each other where these proteins could potentially interact in the form of combinatorial molecular phenotypes (CMPs) in intact cells or tissue^43–46^. A single CMP or groups of CMPs may mediate different cellular functions. TIS has been used to describe localization and potential interaction of multiple proteins in various cells and tissues^43–46^. TIS and toponomics are unique morphological tools in that they consider both levels of expression of multiple markers and the molecular patterns that may result from their potential interaction (CMPs), rather than focusing on levels of a single marker or several individual markers. This is important because many proteins function as components of a supramolecular complex of several proteins working together. Numerous pathway diagrams in the scientific literature illustrate this. The LPS/CD14/TLR4/MyD88-mediated LPS receptor pathway is a particularly good example^47^.

We have previously devised analytical procedures to enable the comparison of TIS data (i.e. distribution of multiple proteins or markers) in a number of cells from different individuals^29,30^. In the published studies we examined the AM toponome from male SP-A KO mice following their “rescue” with exogenous SP-A1^29^, and from humanized transgenic mouse strains expressing either SP-A1 or SP-A2 and AM from SP-A KO mice^30^. TIS, like many other “-omics” technologies is a descriptive, hypothesis-generating tool, although it could be used to test specific hypotheses in conjunction with appropriately designed experimental protocols.

In the present study employing TIS we investigated: 1) the SP-A KO AM toponome from female mice with and without rescue with a single dose of exogenous SP-A1; and 2) compared the AM toponome from female mice (present study) with that of male mice^29^ to assess potential sex differences in their CMPs.

## Methods

The experimental protocol for this study was nearly identical to that of a study we published previously^29^, but is repeated here for convenience.

### Animals

We used female SP-A KO mice that had been generated on the C57BL6/J genetic background. We propagated and raised the mice in our breeding colony at the Penn State College of Medicine and used them for experiments at 8–12 weeks of age. The mice were raised under pathogen-free conditions or in barrier facilities and had free access to food and water. There were sentinel animals housed in the same animal rooms that showed no evidence of respiratory pathogens. This protocol was approved by the Institutional Animal Care and Use Committee of the Penn State College of Medicine. The study design is in accordance with ARRIVE guidelines.

### Treatment with exogenous SP-A1

Mice were anesthetized by injection with Ketamine (Ketaject, Phoenix Pharmaceuticals Inc., St. Joseph, MO) and Xylazine (XYLA-JECT, Phoenix Pharmaceuticals Inc., St. Joseph, MO) prior to administration of either vehicle or an aliquot of vehicle containing SP-A1. The SP-A1 was isolated from stably transfected CHO cells and purified using mannose affinity chromatography as described previously^48^. SP-A1 preparations were made with the SP-A1 6A^2^ variant. This SP-A1 variant occurs in the general population with the greatest frequency^8,49^. The exogenous SP-A1 treatment contained SP-A1 (10 μg) in 50 μl of sterile saline with 1 mM CaCl_2_. We have used similar doses of exogenous SP-A in previous rescue studies^22,24,29,35,37,38^. Control animals received 50 μl of vehicle (saline and 1 mM CaCl_2_) alone. We suspended the anesthetized mice by their maxillary incisors, placed the bolus containing SP-A1 or vehicle in the pharynx, and briefly blocked the nostrils, resulting in the aspiration of the instilled bolus. After recovering from anesthesia, the mice were returned to their cages until it was time to harvest alveolar macrophages. In previous studies^22^ we have found this method of introducing SP-A and other substances to the lungs to be very consistent and reproducible.

### Sample preparation

As in our previous study^29^, eighteen hours after treatment with either vehicle or SP-A1 the mice were euthanized and underwent bronchoalveolar lavage (BAL) with phosphate-buffered saline (PBS), 1 mM EDTA. The AM obtained were washed and counted. Slides for TIS were prepared by placing a 0.5 mm thick plastic sheet in which a circular opening with a diameter of 8 mm was cut onto a microscope slide. An aliquot with 100,000 cells was placed in the resulting well in a volume of 100 µl of serum-free RPMI medium. The cells were covered with a plastic cap to limit evaporation and the slide was placed in the incubator for 45–60 min to allow the cells to adhere. After the attachment period the slides were gently washed with PBS and then air dried (15 min), immersed in acetone at room temperature (10 s), then in chilled hexanes (− 70 °C) in a methanol/dry ice slush (90 s). The slides were stored at − 80 °C until used for TIS.

In order to perform TIS experiments, each slide was warmed to room temperature. A 1.0 mm thick rubber ring with a diameter of 10 mm that could accommodate a volume of 100 µl was placed over the adherent cells. The cells were rehydrated, incubated with normal goat serum diluted 1:50 with PBS for 1 h to limit non-specific binding of antibodies, and washed repeatedly with PBS. The slide was then placed on the microscope in the TIS chamber and a view field selected.

### Toponome imaging system (TIS)

We have described TIS and the accompanying data analysis in previous publications^29,30^. The system used was the TIS basic 4 (pi4 Robotics GmbH, Berlin, Germany). It consists of a climate-controlled chamber containing: a Zeiss AxioImager microscope with a Colibri.2 lighting system and a Plan-Apochromat 63X/1.0 Ph3 M27 water immersion objective; an SC4022M digital imaging system (Finger Lakes Instrumentation, LLC, Lima, NY); and a robot-controlled motorized pipette. The TIS system included software programs (written by Reyk Hillert, Magdeburg, Germany) that were used to generate data for subsequent analysis. These included: Image Registrator v.1.1 (for image alignment and background subtraction); Binary Center v.1.0.2 (for binarization of images); MoPPi v.1.1.3.8 (converts binarized .pgn files into a single .xml file); and MultiCompare v.0.9.0 (extracts CMP data from .xml files). A flow chart for TIS image analysis is shown in Supplementary Fig. S1.

### Antibody calibration/optimization

**Figure 1:**
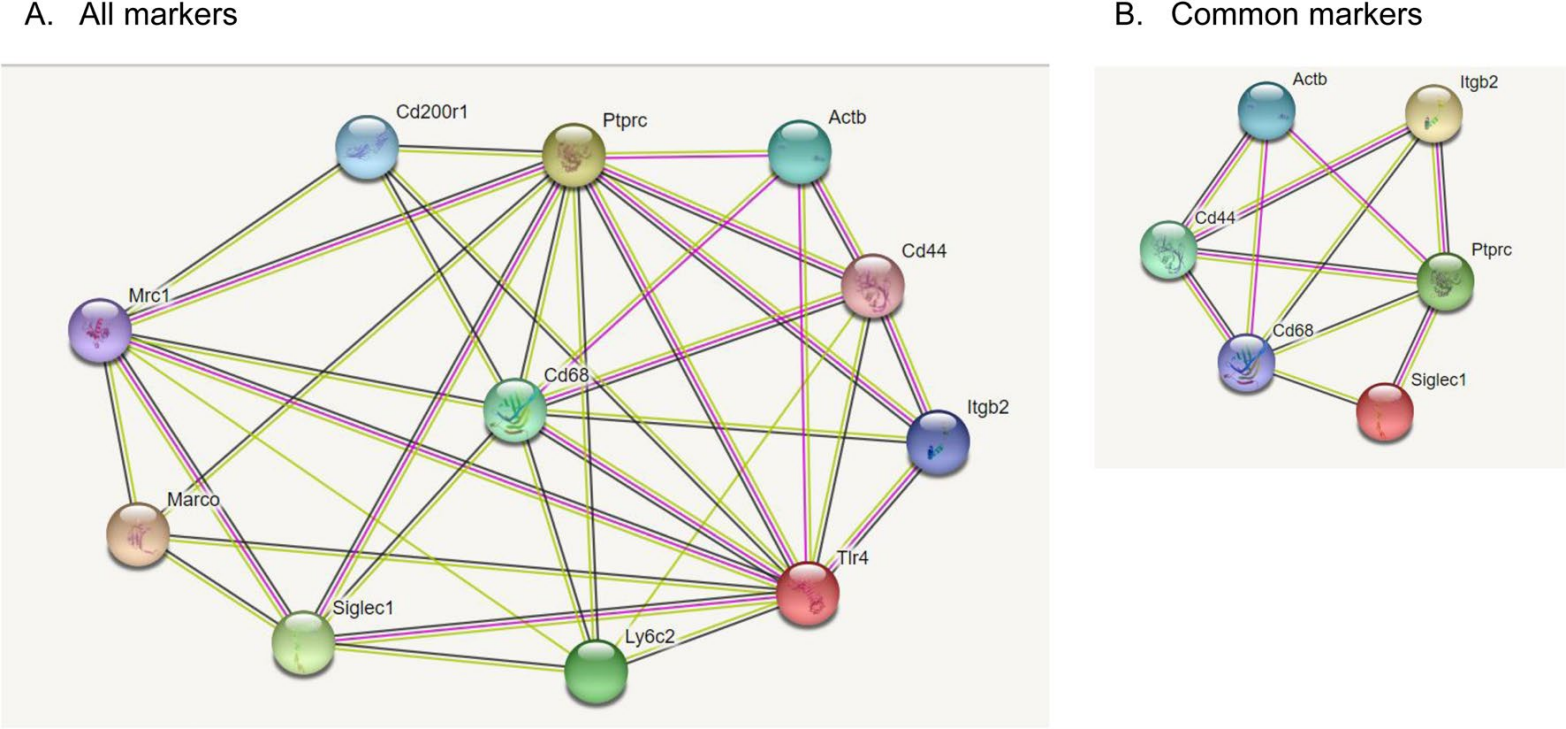
Interaction Diagrams. (**A**) used the gene names for the markers (with the exception of Marker 0 (cellular autofluorescence), which were entered into the String database (string-db.org) to generate an interaction diagram. The gene name for β-actin (actb) was used as a surrogate for phalloidin. (**B**) We entered 6 markers found in common in both this study and a similar study^29^ involving male mice (indicated by (★)) to generate a similar diagram.

All reagents (antibodies and phalloidin) were conjugated with fluorescein isothiocyanate (FITC) and commercially obtained (Table 1). Optimization of antibodies for TIS included determination of appropriate antibody dilution and exposure time for imaging, as described previously^29^. An incubation time of 30 min was used with all antibodies. After optimization, TIS runs were set up with the whole series of antibodies. The TIS procedure is summarized in a flow chart (Supplementary Fig. S1). After imaging, bound FITC-conjugated reagents were photobleached, the sample was re-imaged, and the second image was used for background subtraction during image processing. The photo-bleached slide was then subjected to another round of immunostaining with the next marker. Table 1 lists the antibodies used, their gene names (where appropriate), Uniprot accession numbers, source of antibody, and catalog number of each antibody. The TIS software labels the first marker used as marker #0. That numbering scheme will be used in all figures, tables, and the text. Markers also used in our published study of SP-A KO AM rescue in males^29^ are marked with a “star” in Table 1. Potential interactions between the markers used are shown in an interaction diagram (Fig. 1) generated by the String database (https://string-db.org).

**Table 1:** Markers for female rescue and sex comparison study.

Common (also in male)	Marker # (# for comparison)	Marker or protein name	Accession #	Gene name	Supplier	Catalog #
	0 (0)	Cellular autofluorescence	–	–	–	–
	1 (1)	Sialoadhesin (Siglec 1; CD169)	Q62230	Siglec1	Bioss	bs-10751R-FITC
	2 (2)	CD44, Pgp-1, H_CAM, Ly-24	P15379	Cd44	BD Pharmingen	553133
	3	CD200R (Mox-2; Ox-2)	O54901	Cd200r1	ThermoFisher	MA5-17984
	4	CD206 (mannose receptor; C type 1)	Q61830	Mrc1	ThermoFisher	MA5-16870
★	5 (3)	CD68 (macrosialin)	P31996	Cd68	ThermoFisher	MA5-16676
	6	MARCO	Q60754	Marco	ThermoFisher	MA1-80183
★	7 (4)	CD45 (Receptor-type tyrosine-protein phosphatase C	P06800	Ptprc	BD Pharmingen	553080
	8 (5)	CD18 (LFA-1, Mac-1) Integrin B2	P11835	Itgbb2	BD Pharmingen	553292
	9	Toll-like receptor 4 (TLR4 ), CD284	Q9QUK6	Tlr4	ThermoFisher	MA5-16212
	10	Lymphocyte antigen-6c2 (Ly-6C2; Ly-6C)	P0CW03	Ly6c2 (Ly6c)	BD Pharmingen	553104
★	11 (6)	Phalloidon	–	–	ThermoFisher	F432

List of markers: The markers used in this study are listed together with the accession number, gene name, supplier, and catalog number. The TIS software automatically assigns the first marker as marker #0. Markers that were in common with male rescue study^29^ are indicated (★) and the numbers assigned for comparison (# for comparison) are indicated in parentheses.

### Image processing for TIS

#### Whole image analysis

Whole images contained 2048 × 2048 pixels (although a 15-pixel margin around the periphery of each image was not included) and covered an area on the slide of 117 nm × 117 nm. The steps used to take the original immunofluorescence images depicting the localization of each marker and convert it into a merged data file containing the aggregate data for the localization of all markers in the experiment have been described in detail previously^29,30^ and will not be repeated here. Key steps in that process are presented as Supplementary Material (see Supplementary Fig. S1). The merged data set compiled for the whole image was then converted into SAS (Version 9.4) for subsequent statistical analysis.

#### Analysis of whole images

**Figure 2:**
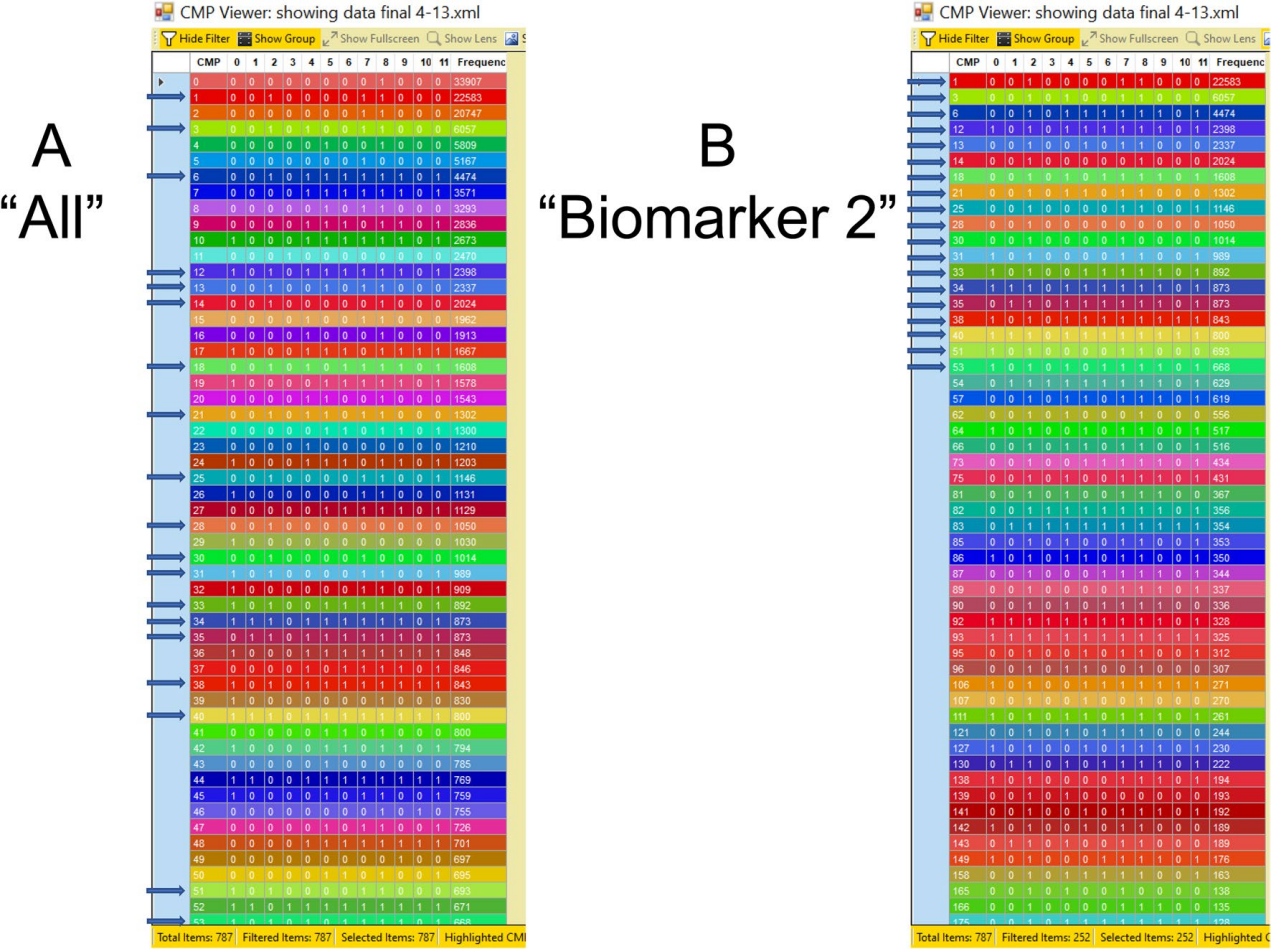
CMP tables. Representative CMP tables are shown from one of the samples in this study. (**A**) The 54 most abundant CMPs in the sample. The CMPs are numbered consecutively (first column on left) based on frequency (abundance; number of pixels) shown in last column. The TIS software automatically assigns the first marker as marker #0 and the most abundant CMP as number “0”. Note that column 0 (for marker 0) contains a mixture of 0s and 1s depending on the absence or presence of that marker in each CMP. Intervening columns show whether each of the markers is present (1) or absent (0) in each CMP. All of the CMPs with a “1” for marker 2 are marked with an arrow. (**B**) A similar table in which only CMPs containing marker 2 are shown. Note that for column 2 all entries show a “1”, indicating the presence of marker 2. Also, note that the CMP numbers are no longer consecutive, but list the 54 most abundant CMPs containing that marker. The first 19 CMPs are marked with an arrow and correspond to the CMPs marked with arrows in (**A**). Similar marker-specific tables were generated for each marker. Tables like this have been described previously^29,30^.

To describe data from whole images (those containing all cells from a given sample) we used a previously described method^29^ where one can assess how similar the samples (n = 3) from each group studied (Vehicle and SP-A1 rescue) are to each other. These comparisons, in the present study, were focused on the 54 most abundant CMPs from each image. The initial step in this analysis is to use tables compiled from all of the CMPs (Fig. 2, Panel A), and then, by going through the list, to identify CMPs that were among the most abundant CMPs for each of the three samples in each group. As stated above, the TIS software automatically designates the first marker as #0, and assigns the most abundant CMP an identifying number of “0”.

The next step was to use a utility in the TIS software that allowed us to select only the CMPs containing a given marker and generate marker-specific tables as shown in Fig. 2, Panel B for marker 2. These marker-specific tables served as data sets for further analysis as follows. We first considered only CMPs with exact matches of all 12 markers. The digital representation of each CMP (i.e. the 12-digit representation of present (1) and absent (0) markers) was used rather than the ranking assigned by the TIS software (CMP#; Fig. 2, Panel A) based on abundance. For example, CMP 0 (the highest frequency) in Fig. 2, Panel A was designated 000000001000 based on the presence (1) or absence (0) of all markers after binarization. A similar approach was used in the sex comparison study involving 7 markers (used in common in both sexes) that is described below. When each of these 12-digit representations of each CMP were compared among samples and found to be present in the 54 most abundant CMPs in all members of a studied group (3-of-3) we considered them to be “conserved” CMPs. We next extended this comparison to include CMPs that were present in 2-of-3 samples as a less stringent measure of conservation. This type of CMP comparison was also subsequently done using the marker-specific CMP tables. An example a marker-specific table is shown in Fig. 2, Panel B for marker 2.

It should be noted that because frequency (abundance) in the whole image is highly dependent on the number of cells in each image (which varied widely) it was not a useful quantitative parameter for comparing whole images to one another for analytical purposes.

#### Single cell analysis

**Figure 3:**
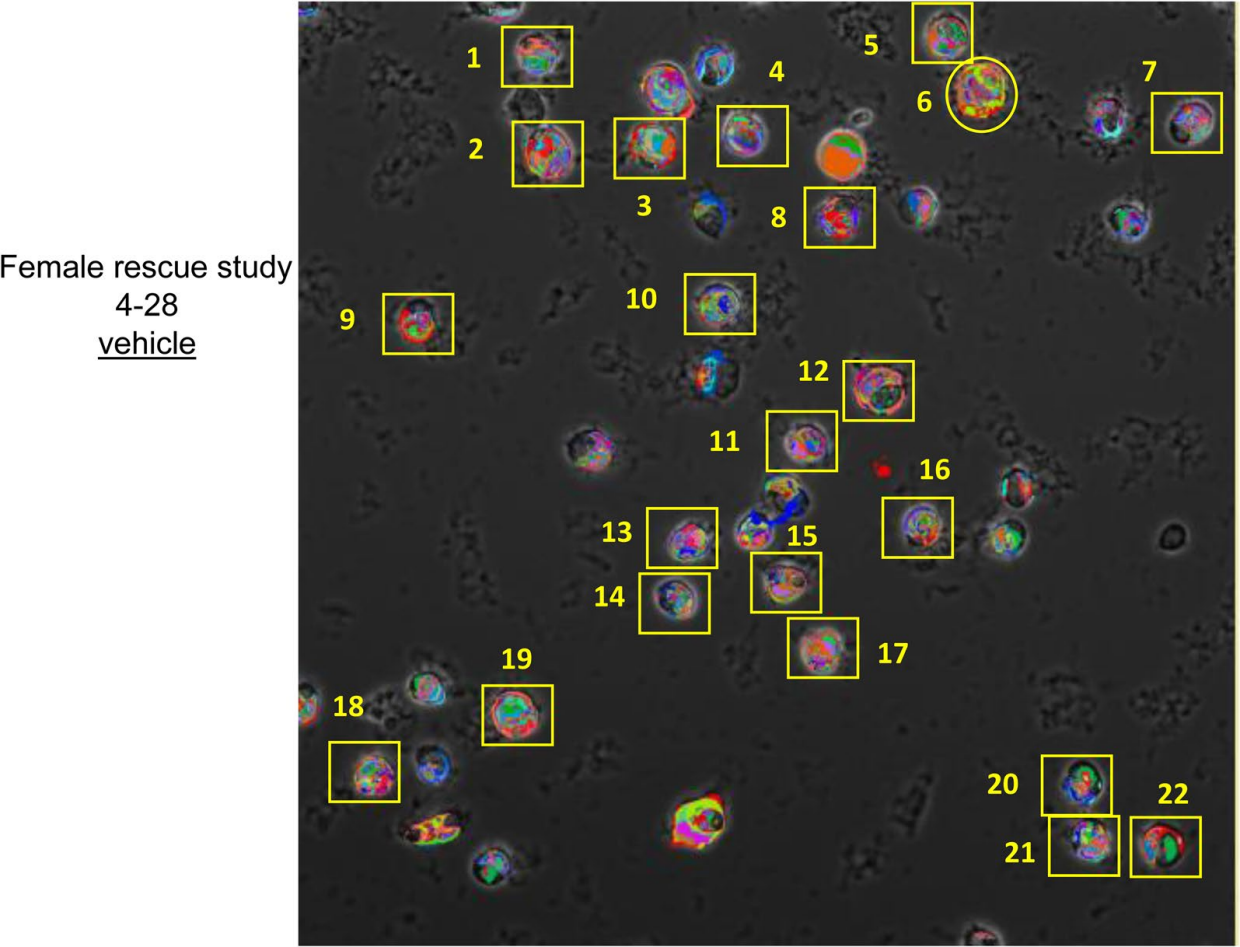
Selected cells. A representative image of one sample in this study is shown. Pseudocolors have been assigned by the TIS software to indicate different CMPs. The outlined cells (n = 22) have been selected and their coordinates determined for single cell analysis.

Individual cells from each image were analyzed instead of the whole image analysis described above. The selected single cells were grossly normal in appearance and did not touch any other cell (Fig. 3). The pixel address for each chosen cell was determined using Image J software 1.53a (https://imagej.nih.gov/ij/download.html) and then the pixel address was used to extract the corresponding pixels from the data set for that image (Supplementary Fig. S1).

#### Summary of CMP content of a given cell

To provide a summary of the CMP content of a given cell we tabulated the number of times a given marker appears in the 20 most abundant CMPs of that cell. We have described the basis of this method in previous TIS publications^29,30^ and it is shown in Supplementary Fig. S2. Briefly, Panel A of the figure depicts a table showing the presence or absence of each marker in the 20 most abundant CMPs for a single cell. The bottom line of the table contains the sum of the number of times each of the 12 markers appears in the top 20 CMPs. For example, marker 1 is present in 10 of the top 20 CMPs. The data in this row of the table are used to produce a line graph summarizing the composition of that cell as shown in Panel B. This was done for all of the selected cells in each image.

#### Hierarchical clustering analysis

**Figure 4:**
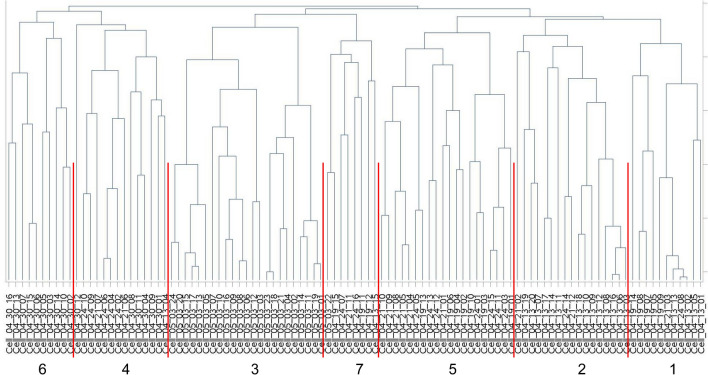
Clustering analysis of female AM. A clustering analysis of the female AM (n = 103 cells) was performed using SAS, Version 9.4 and the resulting dendrogram is shown. The clusters have been delineated with the red lines and are numbered. Cluster numbers are assigned by the statistical program.

**Figure 5:**
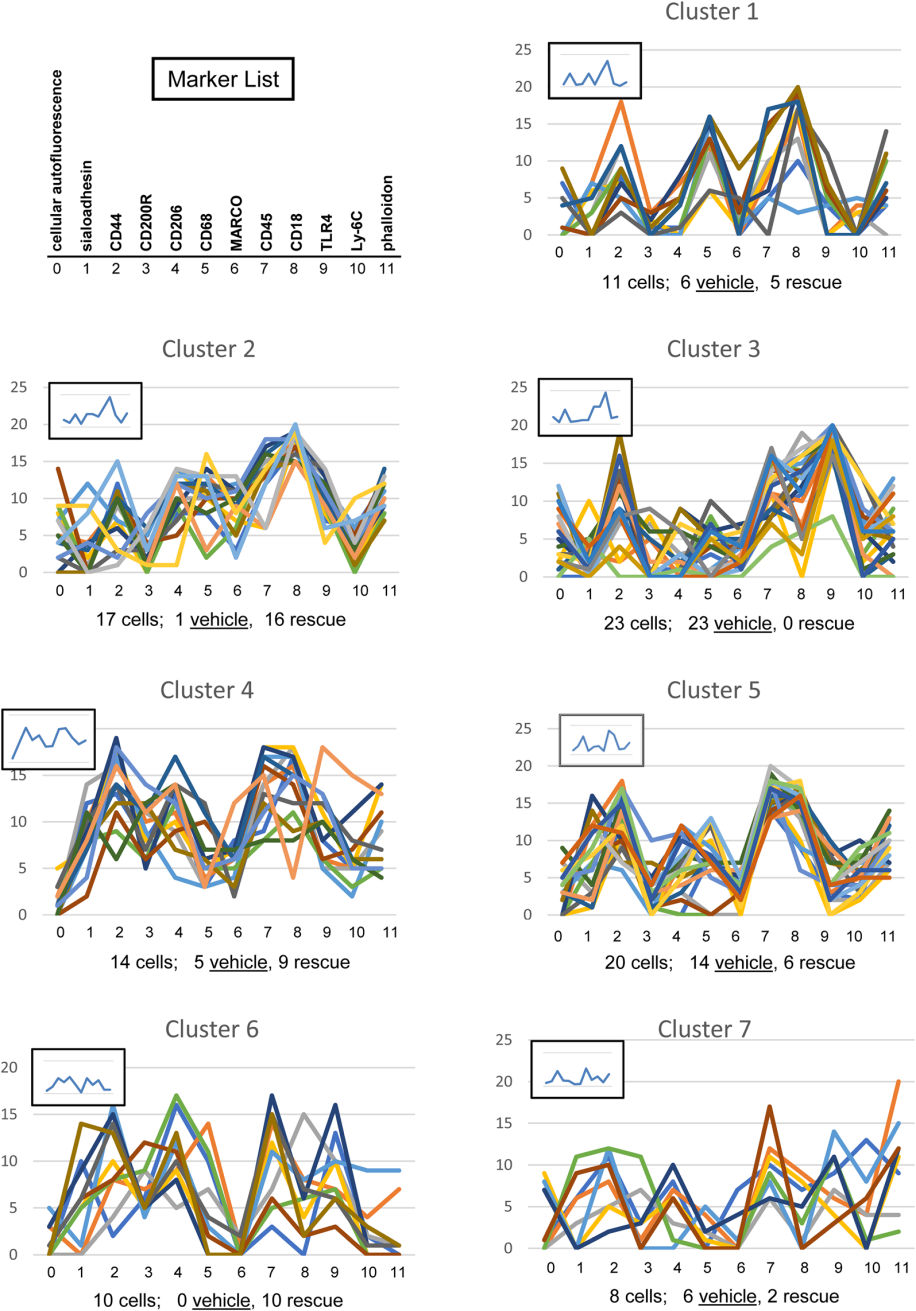
Graphs of cells in each female cluster. The CMP summary of all cells in a given cluster (from Fig. 4) is shown from the 103 AM from female mice. The first panel shows a marker list equating each marker number with a specific protein. Each line represents a single cell and the summary statistics indicating total cell numbers and number of vehicle- and SP-A-treated cells in each cluster are given below each graph. The inset shows a graph summarizing all cells in the cluster based on the mean values of all markers in all cells.

The CMP data obtained from all of the selected cells were then used to perform hierarchical clustering analyses and the results were presented as a dendrogram (Fig. 4). A graphic CMP representation of all cells in each cluster was subsequently generated using the method described above (Fig. 5; Supplementary Fig. S2)^29,30^.

#### Cluster scoring system

A scoring system was devised to categorize the constituent cells of the individual clusters produced in the different clustering analyses. In a given cluster, if the number of cells from the Vehicle group was equal to or greater than 67% of the total number of cells in that cluster, we considered the cluster to be “predominantly Vehicle.” Conversely, if 33% or fewer of the cells in the cluster were from the Vehicle group, we considered the cluster to be “predominantly SP-A1 rescue.” If the number of cells in the Vehicle group fell between these two limits, we considered the cluster “mixed.” These criteria were used to characterize cell populations delineated by each cluster.

The summary scoring method described above was expanded for the sex comparison to include sex. Namely, if a cluster contained 67% or more cells from females, we considered it to be “predominantly female” and if it contained fewer than 33% female cells, we considered it “predominantly male.” If the number of females in a cluster was between 33 and 67% the cluster was considered “mixed” with respect to sex.

#### Sex comparison

To determine whether there were sex differences in the response to SP-A1, we generated merged files for the AM from female mice that included only the 7 markers that were also included in a similar rescue study we had performed previously with AM from male mice^29^. For the comparison we only examined the single cell data. The data sets were created by taking the subset of fluorescence images for specific markers that were in common between the female (present study) and male^29^ rescue studies and generating new merged images and data files (*.xml files) for the 7 common markers. As with the 12-marker data set, the xml files were then read into SAS for further analysis. The data for the 7 markers within the cells were quantified via the graphic CMP representation method we described above. Hierarchical cluster analysis was applied to determine cells with similar characteristics according to the 7 markers (Supplementary Fig. S2). Qualitative and quantitative assessments of the clusters were pursued for the purpose of identifying sex differences.

### Ethics approval

This study was approved by the Institutional Animal Care and Use Committee of the Penn State College of Medicine.

## Results

### Markers and interactions

Table 1 contains a list of the markers (names, accession numbers, gene names, supplier, and catalog numbers) used for this study in which female SP-A knockout mice were “rescued” with an intratracheal dose of exogenous SP-A1 or with vehicle. A total of 12 markers were used. The first of these, as in our previous TIS studies^29,30^, was cellular autofluorescence (AF) of AM. AF, which has been shown to be heterogeneous in AM, may be useful for the analysis of myeloid cells^50^. The intracytoplasmic localization of AF may indicate that potential sources for the AF include NAD(P)H, flavins, ceroid/lipofuscin, bilirubin, porphyrins, among others^50^. In the present study most of the AF was punctate or granular in nature, potentially indicating its localization in lysosomes or phagosomes and related to the bactericidal capacity of the cells. It should be noted that this AF was completely eliminated by the standard series of photobleaching cycles. Next, in successive TIS cycles we used 10 different FITC-labeled antibodies that recognized proteins important for macrophage function. Finally, we used FITC-labeled phalloidin, which binds to filamentous actin (F-actin).

Figure 1A shows an interaction diagram for 11 of the 12 markers (AF cannot be attributed to a single gene) prepared using the String database (STRING: functional protein association networks (string-db.org)). The input for this diagram were the names of the 10 mouse genes encoding 10 of the marker proteins and the gene for beta-actin (*actb*), the main constituent of F-actin, which we used as a surrogate for phalloidin. The diagram (Fig. 1A) demonstrates the extensive degree of interaction between the markers. Notably, both CD45 (*Ptprc*) and *Tlr4* each had ten interactions, and *CD68* had nine interactions. This highlights their important roles in macrophage function. The least number of interactions, observed for several markers, was four interactions.

In a previous study using male mice we used a cohort of 13 markers to do a similar characterization of AM^29^. Due to technical issues and changes in the availability of antibodies, there were 7 markers that were used in common in both the present study (females) and the published study (males). These were then used for the comparison of AMs from males and females. The “common” markers are designated in the first column of Table 1. An interaction diagram (Fig. 1B) was made using the five genes whose products were directly examined, as well as *Actb,* as described above. As with the full set of markers (Fig. 1A), there is also a high degree of interaction with the “common” markers, particularly with *CD68,* CD45 (*Ptprc*), and *CD44* (Fig. 1B).

### Toponome of the SP-A KO AM from female mice with and without rescue

#### Conservation of CMPs in whole image analysis

Based on the postulate that CMPs present in multiple members of a group were more likely to represent a distinguishing feature of that group, we sought to tabulate CMPs present in all or most members of a given group. We referred to these CMPs as matches or “conserved CMPs.” The analysis was restricted to the 54 most abundant CMPs from either the full data set containing all CMPs for that image (Table 2, column labeled “All”), or from each of the marker-specific tables listing the 54 most abundant CMPs that contain the marker designated at the top of the columns in Table 2 (as demonstrated in Fig. 2). Figure 2 demonstrates how the TIS software organizes the data from each image and how it facilitates probing each data set for CMPs that contain a specific protein. Panel A is an example of a data set that would be examined for “All,” meaning CMPs defined by all 12 markers. The CMPs are numbered consecutively based on frequency (i.e. abundance; number of pixels in the image). In the column for marker 0 there is a combination of 0s and 1s, indicating the absence or presence of marker 0 in each CMP.

**Table 2:**
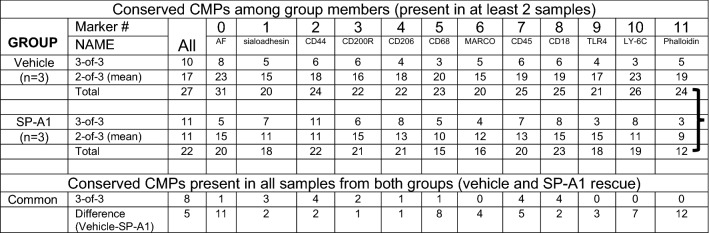
Conservation of CMPs among samples.

In the whole image data, CMPs present in all 3 Vehicle samples (3-of-3) and all 3 SP-A1 rescue samples were tabulated, as well as CMPs that were present in 2 out of 3 of the samples (2-of-3) for each group. The total of the 3-of-3 and 2-of-3 is then given. These are referred to as “conserved CMPs.” The first data column (All) lists the CMPs present in the 54 most abundant CMPs for all 3 samples. Subsequent columns (labeled 0–11) compare CMPs that include the most abundant CMPs for each marker. For example, in the column labeled Marker 0, only the CMPs that included Marker 0 were compared. At the bottom of the table, the row labeled “Common” lists how many of the conserved CMPs are present in both Vehicle and SP-A1 rescue groups. Totals (3-of-3 plus 2-of-3) were compared (indicated by **}**) by the Wilcoxon Rank Sum Test and found to differ significantly (*p* < 0.0004).

By contrast, Panel B, demonstrates a marker-specific data set that lists only CMPs containing marker 2. In the column for marker 2 all entries are 1s, as expected, since these CMPs were selected because they all contain marker 2. The CMPs are still numbered sequentially, but there are gaps in the numbering corresponding to CMPs that did not contain marker 2.

Note that in Panel A all of the CMPs containing marker 2 (n = 19) are marked with an arrow and are scattered among the 54 most abundant CMPs. In Panel B these CMPs are the first 19 among the 54 most abundant CMPs containing marker 2. Because all of the CMPs in this data set contain marker 2, marker 2 is referred to as a “lead protein”^46,51^. Similar marker-specific data sets were generated for each of the markers and probed as described above to assess conservation of CMPs among group members.

We counted CMPs that matched or were present in all three members of the groups (3-of-3), as well as CMPs that matched or were present in two out of the three members of the group (2-of-3), and refer to these as “conserved CMPs.” Table 2 shows the tabulated values for “All” and for each marker. We also recorded the sum of both the 3-of-3 and 2-of-3 CMPs (Total). For example, in the first data column (All) 10 of the 54 most abundant CMPs were found in all three members of the Vehicle group and 17 of the 54 most abundant CMPs were present in two of the three members. This gave a total of 27 CMPs out of the most abundant 54 that were well conserved among the Vehicle group members. The SP-A1 rescue group had 11 CMPs present in all three SP-A1 group members (3-of-3) and 11 present in 2-of-3 group members for a total of 22. The 2-of-3 numbers are derived from making 3 different pairwise comparisons among the three samples (i.e. sample #1 vs sample #2; #1 vs #3; #2 vs #3) to identify conserved CMPs. The mean value of these three comparisons is entered into the table as the 2-of-3 value. For example, in the case of the “all marker” comparison of the SP-A1 cells, sample #1 has eight 2-of-3 matches, sample #2 has fourteen, and sample #3 has eleven. This yields a total of thirty-three 2-of-3 matches for this group and a mean value of eleven 2-of-3 matches per sample (33 matches/3 samples = 11 matches/sample), as indicated in Table 2.

The remaining columns are the CMPs that all contain the marker indicated at the top of the column using the marker-specific data sets (see Fig. 2, Panel B). All of the results for this analysis are compiled in Table 2. The columns for Marker 0 (AF) and Marker 11 (Phalloidin) are particularly notable because the differences between the Vehicle and SP-A1 rescue groups are very large. There were 31 conserved CMPs for AF in the Vehicle group and only 20 for the SP-A1 rescue group and there were 24 conserved CMPs for Phalloidin in the Vehicle group and only 12 in the SP-A1 rescue group.

In all cases there was a higher degree of conservation in the Vehicle group than in the SP-A1 group. The differences were compared (indicated by **}**) with the Wilcoxon Rank Sum Test and found to be significant (*p* < 0.0004). This analysis, showing greater conservation or similarity in the Vehicle group, indicates that of the two groups studied, the Vehicle group is more uniform and the SP-A1 rescue group is more diverse. We also examined the most conserved (3-of-3) across the two groups (i.e. conserved in both Vehicle and SP-A1 rescue groups) and listed the values. In the “All” column we found that 8 CMPs were present in all of the samples of both groups in the study. However, there were relatively few of the marker-specific CMPs that were present in both groups.

#### Single cell analysis

From each whole image, a number of single cells were selected, mapped, and the pixels making up that cell defined. This allowed us to compare CMPs in individual single cells. A representative image from one of the samples in this study is shown in Fig. 3. A total of 103 cells were selected from the 6 samples studied. This total consisted of 48 cells from the SP-A1 rescue group (n = 3 rescue mice) and 55 cells from the Vehicle group (n = 3 vehicle mice). The CMPs from each of these cells were used for cluster analysis.

##### Hierarchical cluster analysis

First, we applied the 12-marker data set to a clustering analysis. Figure 4 is a cluster dendrogram compiled from the 103 cells from the female data set. As mentioned above, these included the CMP data from 48 cells for the SP-A1 rescue group and 55 cells from the Vehicle group. Each group contained cells obtained from three mice. The cluster analysis defined 7 clusters, each of whose composition of vehicle and rescue cells is shown in Table 3. As in the cluster analysis of other datasets that we have reported, the data from the dendrogram depicted in Fig. 4 are summarized in tabular form. Table 3 shows that this unbiased analysis revealed cluster 6 (exclusively) and cluster 2 (16 of 17) to be composed of SP-A1 rescue cells, whereas cluster 3 contained only Vehicle cells. These data indicate that the levels and combinations of markers in the cells of these particular clusters are likely to be dependent on either the presence or absence of SP-A1. The other clusters, most notably clusters 1 and 4, contained cells from both groups demonstrating that cells in these clusters could possess marker combinations independent of the SP-A1 status. We considered each of these clusters to represent a cell phenotype.

**Table 3:** All females—clusters—12 markers.

Composition of each cluster (from Fig. 4)								
Group/cluster #	1	2	3	4	5	6	7	Total
Vehicle	6	1	23	5	14	0	6	55
Rescue	5	16	0	9	6	10	2	48
Total	11	17	23	14	20	10	8	103

Summary of female AM cluster analysis. The female AM data were subjected to cluster analysis (see Fig. 4) and the composition (Vehicle or SP-A1 rescue) of each cluster is shown in this table. When all 103 AM from the female samples were subjected to cluster analysis based on their CMP content, 7 clusters were defined and the makeup of each cluster is shown in this table.

##### Graphing and scoring of individual clusters

The line graphs of the individual cells described above in a given cluster were then plotted to get the “signature” of a given cluster. Figure 5 shows all the graphs (drawn using the method described above and in Supplementary Fig. S2) for all cells in each cluster as defined by the dendrogram in Fig. 4. In the first panel, we show a marker list to expedite the process of equating each marker number with a specific protein. Each line represents a single cell. Below the graphs, we show the categorization of the cells (i.e. total, group, sex) making up each cluster. It is notable that although the graphs for the cells in each cluster share some similarities and may have some peaks or valleys in common, each produces a distinct line and no two cells are identical. For example, we could consider some common features in Cluster 1, such as the peaks for markers 2, 5, and 8 and the valleys for markers 3 and 10, to be characteristic of a phenotype represented by that cluster. In this case, Cluster 1 is almost evenly divided between Vehicle and SP-A1 rescue cells so we could consider this particular phenotype to be independent of the presence of SP-A. The inset in the upper left of each panel is a graph produced by calculating the mean of the marker levels of all cells in the cluster. This calculated marker mean value represents an average value for all of the CMPs in all of the cells in a given cluster.

An example of some characteristics that might differentiate rescue from vehicle cells is seen in the comparison of clusters 2 and 3. The summary graphs (insets) are quite similar for both, but cluster 2 (almost exclusively rescue) shows elevated levels of markers 4, 5, and 6 relative to cluster 3 (exclusively vehicle). Note that these differences are in the relative amounts of the three markers, rather than in their presence or absence. In addition, cluster 2 shows a sharp peak for marker 8 and cluster 3 shows a sharp peak for marker 9.

##### Scoring system

To summarize the clustering analysis, we used the scoring system described in "Methods" section. Using the clustering analysis and these specific scoring criteria to characterize the cell population, we observed that of the 103 total cells: a) 51 cells (or 49.5% of the total cells) were in three clusters that were predominantly Vehicle; b) 27 cells (26.2% of the total) were in two clusters that were “predominantly SP-A1 rescue”; and c) 25 cells (24.3% of the total) were in two clusters designated “mixed.”

### Sex comparison of AM toponomes

We next performed a direct comparison between AM from male and female mice undergoing the same experimental protocol. The 7 markers used in this comparison are indicated with stars (★) in the first column of Table 1 and their theoretical interactions are shown in Fig. 1B. The cells analyzed for the comparison were the same set of female cells characterized above and the set of male cells we described previously^29^. The latter (i.e. male cells) were further characterized by clustering analysis, tabulating the cells in each group, and showing the graphs of the cells in each cluster (Supplementary Figs. S3, S4, Supplementary Table S1). Altogether, there were 217 cells (103 female; 114 male; or 112 Vehicle; 105 SP-A1 rescue) that were compared as follows.

#### Comparison of group marker means

For the comparison of AM from female and male mice the mean amounts of each marker for all of the selected cells for each group were calculated and tabulated as shown in Table 4. These values were analogous to the mean values in the insets in Fig. 5. However, these were generated using all of the cells in the group denoted in the left-hand column of the table, and not only the cells of a given cluster as described in Fig. 5. For example, in the “All” group of the selected single cells, the mean level of marker #1 was 0.15. A series of 42 pairwise comparisons (males vs females; vehicle vs SP-A1 rescue; etc.) was then done for various groups and the resulting *p* values adjusted with the false discovery rate. The pairs that differed significantly (adjusted *p* value < 0.05) are denoted by the brackets in Table 4. With marker #2, for instance, there were significant differences between: (a) all vehicle and all SP-A1 rescue cells; (b) male vehicle and male SP-A1 rescue cells; and (c) female SP-A1 rescue and male SP-A1 rescue cells. These comparisons showed significant differences in all markers except markers #1 and #2. It is noteworthy that there were significant differences between male vehicle and male SP-A1 rescue cells for 4 of the 7 markers, but only 1 significant difference (marker #4) when female vehicle and female SP-A1 rescue cells were compared.

**Table 4:**
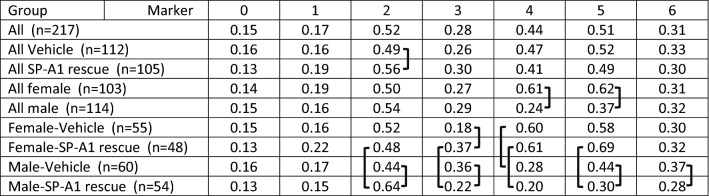
Comparison of group marker means.

For each group indicated in the left-hand column of the table, the number of cells is given. The mean levels of each marker were calculated and listed in the numbered columns. A series of pairwise comparisons were done and significant differences (adjusted *p* value < 0.05) are indicated by the brackets.

#### Hierarchical cluster analysis

**Figure 6:**
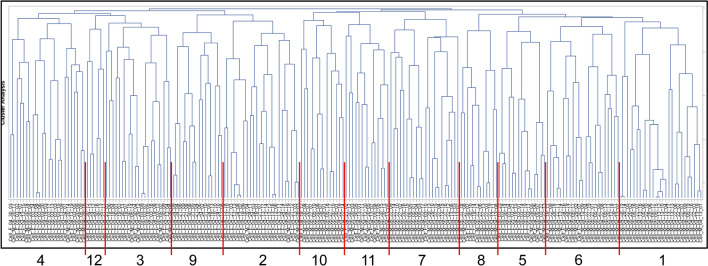
Dendrogram of sex comparison. A clustering analysis of the combined female/male data with the 7 markers in common in both studies (see Table 1) was done using SAS, Version 9.4 and the resulting 12-cluster dendrogram is shown. Cluster numbers are assigned by the statistical program.

**Figure 7:**
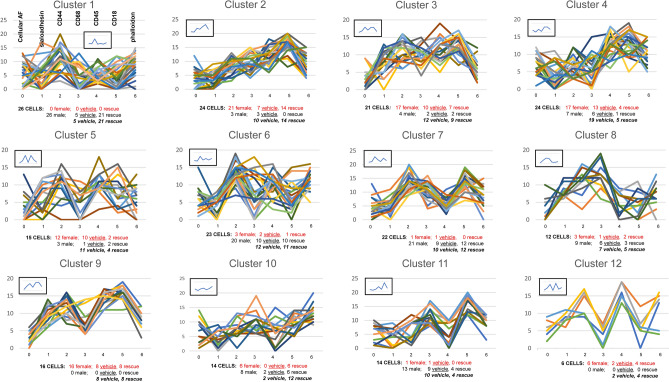
Graphs of sex comparison clusters. The graphs of individual cells in each cluster are shown, together with the calculated marker means graphed in the insets as in Fig. 5. The names of the proteins corresponding to each marker are given in the panel for cluster 1. Below each set of graphs the makeup of each cluster is summarized with respect to sex and experimental group (Vehicle vs. SP-A1 rescue).

The CMP data set was subjected to hierarchical cluster analysis using the data from the 7 markers that were used in both male and female studies, as described above. We selected an analysis that defined 12 clusters (Fig. 6) and their composition is summarized in Table 5. The size of the clusters in this analysis ranged from 6 to 26 cells. The graphs depicting the cells in each cluster are shown in Fig. 7 and the population of each cluster and its details are given below the graph (i.e. Vehicle vs SP-A1 rescue; female vs male). The protein names corresponding to each marker are given above the CMP graphs for cluster 1.

**Table 5:** Both sexes 12 clusters—7 markers (see Fig. 6).

Cluster #													
Group/cluster #	1	2	3	4	5	6	7	8	9	10	11	12	Total
**A. Number of female and male cells in each cluster (from Fig. ****6****)**													
Female	0	21	17	17	12	3	1	3	16	1	6	6	103
Male	26	3	4	7	3	20	21	9	0	13	8	0	114
Total	26	24	21	24	15	23	22	12	16	14	14	6	217
**B. Number of Vehicle and SP-A1 rescued cells in each cluster (from Fig. ****6****)**													
Vehicle	5	10	12	19	11	12	10	7	8	10	2	2	112
SP-A1	21	14	9	5	4	11	12	5	8	4	12	4	105
Total	26	24	21	24	15	23	22	12	16	14	14	6	217

The table summarizes the details of a cluster analysis shown in Fig. 6 that includes the 217 cells used in this study. In Part A the number of cells in each cluster from male or female mice are listed. In Part B the cells making up each cluster are described by whether they are from Vehicle-treated or SP-A1-treated mice.

When the clusters were examined for their ability to differentiate Vehicle and SP-A1 rescue three of the 12 clusters containing 53 cells (24.4% of total cells) were “predominantly Vehicle”, and another three clusters containing 46 cells (21.2%) were “predominantly SP-A1 rescue”, and six clusters containing 118 cells (54.4%) were “mixed” with respect to Vehicle and SP-A1 rescue. When we categorized the clusters based on sex, we found that six clusters containing 106 cells (48.8% of total cells) were “predominantly female,” and five clusters containing 97 cells (44.7%) were “predominantly male.” Only a single cluster with 14 cells (6.5%) was classified as “mixed.” These results indicate that with the number of markers studied here (n = 7) the clustering analysis of CMPs was more effective in delineating clusters on the basis of sex compared to its ability to distinguish between experimental groups (Vehicle or SP-A1 rescue). This was borne out by statistical analysis. The data in Table 4 were used to calculate Cramer’s V (0.75 for sex, and 0.39 for experimental group), a measure of association between two nominal variables. The results confirmed that the 12 defined clusters are more highly associated with sex than with treatment group.

## Discussion

The involvement of SP-A in innate immunity has been extensively documented^4–7,10,12^. Our laboratory has used two approaches to investigate this important action. On one hand, we have studied the cumulative effects of SP-A on innate immunity by investigating the improvement in survival of mice in a pneumonia model in the presence or absence of ozone-induced oxidative stress^34,35,39,40^. On the other hand, we have studied molecular mechanisms that are likely to be responsible for the improvement in survival. This was done by studying the effects of SP-A on AM function in terms of cytokine production, gene expression, phagocytosis, the AM proteome, miRNome, and toponome^4,10,13–16,19–30^. In the course of these studies we made two important observations. First, we discovered that SP-A1 and SP-A2 variants differentially affect both survival^10,35,52^ and all of the other endpoints we have examined^11,23,25–28,30,37,38,41^. Second, we found that there are sex differences in the SP-A effects^4,10,24–28,34,35,38–41^. The latter is the focus of this study.

In the present study female mice lacking SP-A were “rescued” with a single dose of exogenous SP-A1 that approximated levels found in WT mice^24,37^ and has been previously used in rescue studies^24,35,37^. The effect of this rescue treatment on the combinatorial expression of 12 different markers in the female AM was investigated via the use of toponomics. A similar study with AM from male mice has been previously published^29^. In the male study there were 13 different markers used, 7 of which were identical to the markers used in the present study with females, allowing us to do a direct comparison between males and females. It should be noted that we used TIS in this study as a “discovery” or hypothesis-generating tool to identify CMP-based phenotypes and potential targets for future study. More focused experimental protocols could use TIS to test various hypotheses.

### Female AM toponome

The toponomic analysis of female AM confirmed several observations we reported previously with AM from male mice^29,30^). Namely, AM are extremely heterogeneous and of the 103 AM studied none were identical. Based on the conservation of CMPs in AM, AM from KO mice were more uniform than those that underwent the SP-A1 rescue. The same trend was observed in the study of male AM. One possible interpretation of this finding is that in the absence of SP-A, a strong differentiating influence on the AM, many of the cells assume a more “primitive” or less differentiated phenotype, as suggested by the increased degree of conservation of CMPs in the vehicle-treated KO AM. Following SP-A1 rescue, these “primitive” cells may differentiate along several different pathways to subserve various AM functions, shown previously and described above, to be regulated by SP-A^4,10^, and this putative SP-A1-mediated AM differentiation is shown here by a reduction in the conservation of CMPs. Moreover, consistent with the CMP conservation analysis, are the observations made in the present study by the unsupervised clustering analysis. Two thirds of the AM from vehicle-treated mice were found in two of the seven clusters defined by the clustering analysis, whereas most of the AM from SP-A1 rescued mice were dispersed over five of the clusters indicating an increased CMP diversity in response to SP-A1.

One interesting example is provided by comparing the summary graphs of two clusters (Fig. 5, clusters 2 and 3). The graphs of the two clusters appear similar, perhaps indicating similar phenotypes. However, one noticeable difference between the two clusters lies in the relative amounts of CD206, CD68, and MARCO (markers 4, 5, and 6). These proteins have known roles in innate immune processes and are relatively higher in the SP-A1 rescue cells. A second pronounced difference is that cluster 2 (mostly rescue) has a peak with marker 8 (CD18), whereas cluster 3 (all vehicle) has a peak with marker 9 (TLR4). These differences are consistent with an enhanced phagocytic capability in the SP-A1 rescue cells versus the well documented deficiencies in KO AM.

The collective toponomic observations indicate an important role for SP-A in the composition of protein clusters or CMPs that, in turn, may contribute to various AM functions. There are examples in the literature that protein or receptor function is often dependent on the presence of a multi-protein complex and that a single protein by itself may not perform that function. The multi-protein complex associated with CD14 provides an example of this^47^. Another example of protein networks is seen using TIS to study rhabdomyosarcoma tumor cells. It was shown that cells in an “exploratory” state^46^ develop extensions that contain a number of CMPs with CD13 as a lead or key protein. When CD13 was blocked, the formation of these cell extensions was inhibited and cell structure (and presumably function) was altered^46^. A lead protein is believed to be responsible for the integrity of a given CMP or group of CMPs^46,51^ and elimination (or neutralization) of that protein can result in CMP disruption and in a compromise of the CMP-mediated function. It is likely that future studies with TIS will provide additional evidence where cell structure and/or function can be altered by inhibiting a lead protein, making these proteins potential therapeutic targets.

### Sex differences

Sex differences between AM at baseline levels or in response to various insults either to bacterial infection and/or ozone-induced oxidative stress have been shown previously^4,10,24–28,34,35,38–42^. Sex hormones appear to play a role in mouse survival after infection in the presence or absence of ozone-induced oxidative stress^39^, and in the AM miRNome after ozone-induced oxidative stress^26^. However, this is the first study to show that AM molecular protein patterns, as defined by CMPs, differ between sexes, as assessed by either individual CMP analysis or CMP cluster analysis. Furthermore, CMP clustering analysis showed that CMPs were more conserved in females (Table 2) than in males^29^, indicating a lower phenotypic diversity in females than in males, and pointing to a more vigorous response to SP-A1 in males than females. This, in turn, is consistent with a proteomics study where SP-A KO AM were rescued with SP-A. Although fewer overall protein changes were observed in females than males in response to SP-A, different groups of proteins exhibited more changes in females or males^24^, indicating that different underlying sex-specific processes may contribute to previously observed sex-specific AM functions^4,10^. However, this more vigorous CMP-based response in males may not be necessarily more advantageous for the well-being of the AM or the organism. A study in an animal model expressing the SP-A2 transgene showed that the AM mitochondrial reactive oxidant species level was significantly higher in males than in females after ozone-induced oxidative stress^42^. On the other hand, bacterial infection resulted in a better survival outcome in females than in males^34,35,40^ and gonadectomy eliminated (females) or significantly reduced (males) survival differences between control and infected animals, whereas sex hormone replacement restored the survival differences^39^.

A direct comparison of CMPs of male and female AM, via the tabulation of the mean levels of each marker in all of the cells (Table 4), revealed: a) more significant differences between Vehicle- and SP-A1 rescue-treated AM from male than from female mice, providing further support that males may undergo a more vigorous response to SP-A1 treatment than females; and b) more significant male/female differences were observed than vehicle/SP-A1 rescue differences. Moreover, the CMP cluster analysis of 217 male and female cells defined by 12 clusters (Figs. 6, 7), was more effective in discriminating AM between males and females than it was between vehicle- and SP-A1 rescue-treated AM, indicating that sex differences may be more pronounced than differences due to treatment. Differences between male and female Vehicle AM (marker 4) and between male and female SP-A1 rescue AM (markers 2, 3, 4, 5), shown in Table 4 were also apparent in the graphs of AM in some of the clusters (i.e. higher levels of marker 4 are observed in some of the predominantly female clusters) (Fig. 7). As expected, these trends were not universal among the groups, but did occur in substantial subpopulations of each cluster. This is in line with observations made in previous TIS studies^29,30^, i.e. phenotypes are rarely exclusive to one group or another although they may be highly enriched.

Although TIS can provide information about the co-localization of various markers as CMPs, one cannot yet directly attribute a function to a given CMP. However, based on the knowledge of individual proteins it may be possible to postulate potential functional implications resulting from increases or decreases of certain CMPs. In this context TIS could provide a foundation for subsequent physiological studies.

## Conclusions

In the present study we showed: (a) an extensive heterogeneity of AM in females, as shown previously in males, as well as the utility of clustering analysis as an analytical tool; (b) AM from female mice lacking SP-A, as with males, have a pronounced response to treatment with exogenous SP-A1 resulting in increased phenotypic diversity; and (c) a direct comparison of the responses of male and female AM to SP-A1 treatment revealed that males respond more vigorously to exogenous SP-A1 treatment than females do. This study, to the best of our knowledge, provides evidence for the first time that AM sex differences may be mediated via differences of CMPs (i.e. molecular protein patterns). The findings here provide insight into the SP-A-mediated sex differences of the alveolar macrophage observed in several previous studies. They also indicate the importance of innate immune molecules in sex-specific function of the alveolar macrophage, and provide a strong foundation for future mechanistic studies.

## Supplementary Information

Supplementary Information.

## Data Availability

The datasets used and/or analyzed during the current study are available on reasonable request.

## Abbreviations

AF: Autofluorescence
AM: Alveolar macrophage
CMP: Combinatorial molecular phenotype
FITC: Fluorescein isothiocyanate
KO: Knockout
LPS: Lipopolysaccharide
NF-κB: Nuclear factor kappa-light-chain-enhancer of activated B cells
PBS: Phosphate-buffered saline
SP-A: Surfactant protein A
TIS: Toponome imaging system
TLR2: Toll-like receptor 2
TLR4: Toll-like receptor 4
