# Supplementary material for: The alveolar macrophage toponome of female SP-A knockout mice differs from that of males before and after SP-A1 rescue
Source: Sci Rep. 2022 Mar 23;12:5039. doi: 10.1038/s41598-022-08114-2 (PMC8943067; doi:10.1038/s41598-022-08114-2)
Supplement: Supplementary file 1 — Supplementary Information. [file 41598_2022_8114_MOESM1_ESM.pdf]

SUPPLEMENTARY MATERIAL

The alveolar macrophage toponome of female SP-A knockout mice differs from that of males before and after SP-A1 rescue.

David S. Phelps, Vernon M. Chinchilli, Lili Yang, Debra Shearer, Xuesheng Zhang, and Joanna Floros

Supplementary Figure S1

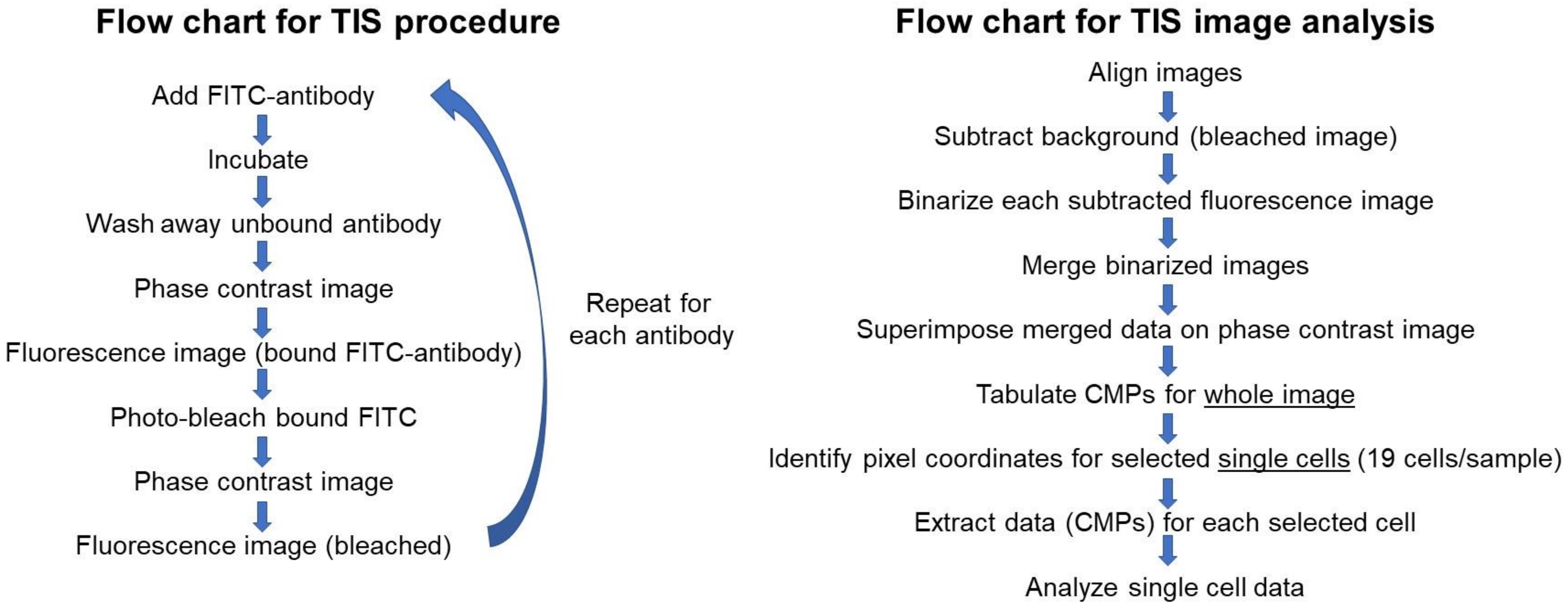

TIS flow chart. A flow chart is shown depicting the steps to conduct the TIS procedure and subsequent data analysis. These procedures have been described in detail previously (29;30).

Supplementary Figure S2

A

Marker number												
CMP #	0	1	2	3	4	5	6	7	8	9	10	11
1	0	0	0	0	1	0	0	0	0	0	0	0
2	0	0	1	0	0	0	0	0	0	0	0	0
3	0	1	0	0	1	1	0	0	0	1	0	0
4	0	0	0	0	1	1	0	0	0	0	0	0
5	0	0	0	0	1	1	0	0	0	1	0	0
6	0	1	0	0	1	0	0	0	0	1	0	0
7	0	0	0	0	0	0	0	0	0	1	0	0
8	0	0	0	1	1	0	0	0	0	0	0	0
9	0	0	0	0	1	0	0	0	0	1	0	0
10	0	1	0	1	1	1	0	0	0	1	0	0
11	0	0	0	0	0	1	0	0	0	0	0	0
12	0	1	0	0	1	0	0	0	0	0	0	0
13	0	1	0	0	1	1	0	0	0	1	1	0
14	0	1	0	0	1	1	0	1	0	1	0	0
15	0	0	0	0	1	0	0	1	0	1	0	0
16	0	1	1	0	1	1	0	0	0	1	0	0
17	0	1	0	1	1	0	0	0	0	1	0	0
18	0	1	0	1	1	1	0	1	0	1	0	0
19	0	1	0	1	1	1	0	0	0	1	1	0
20	0	0	0	1	0	0	0	0	0	0	0	0
Total	0	10	2	6	16	10	0	3	0	13	2	0

B

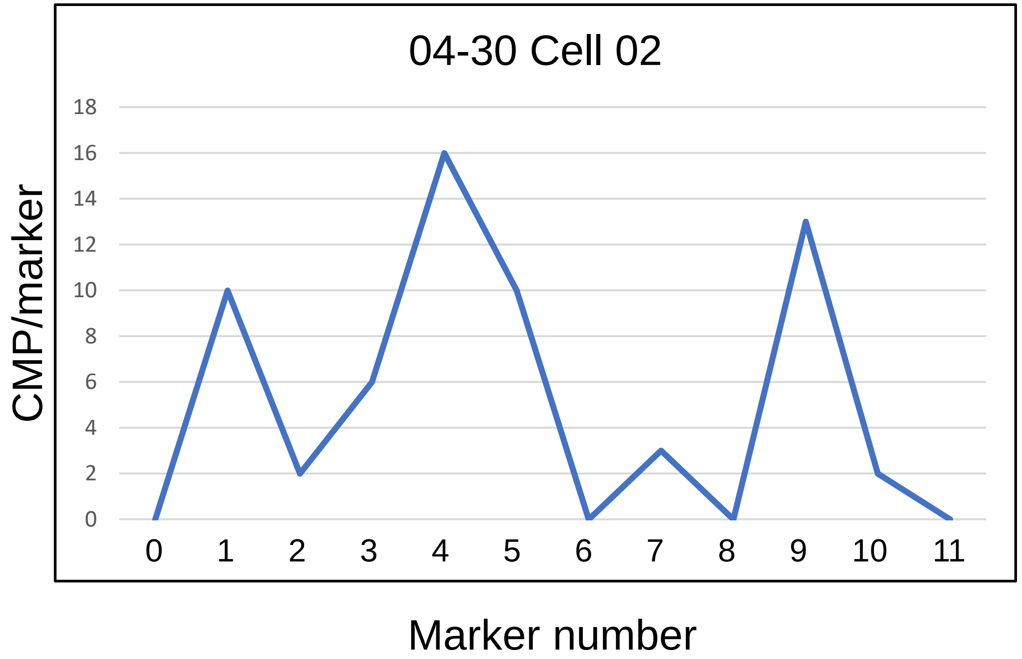

CMP summary graphs. The procedure we have devised for summarizing a cell's CMP content in order to obtain a graphic "signature" for each cell is shown. Panel A shows a table listing the content of the 20 most abundant CMPs for a single cell. The bottom line of the table gives the total number of CMPs (out of the top 20) that contain each marker. The totals are used to prepare the graph shown in Panel B.

Supplementary Figure S3

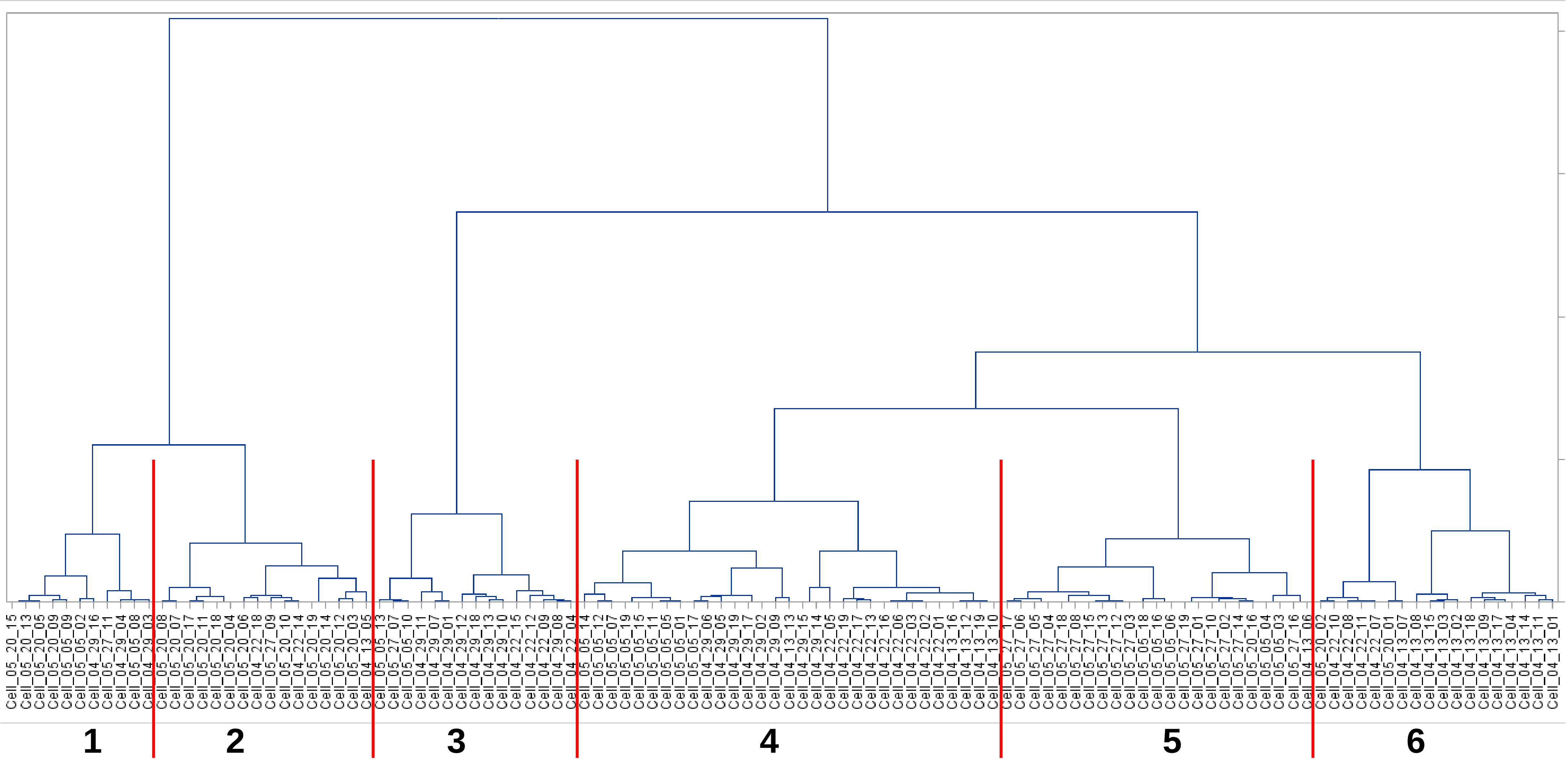

Clustering analysis of male cells. Data for AM obtained from male mice (n=114 cells) described previously (29) that underwent the same experimental protocol as the females in the present study were mined for clustering analysis. The resulting dendrogram is shown here with the 6 clusters delineated with red lines. Cluster numbers are assigned by the statistical program.

SUPPLEMENTARY FIGURE S4

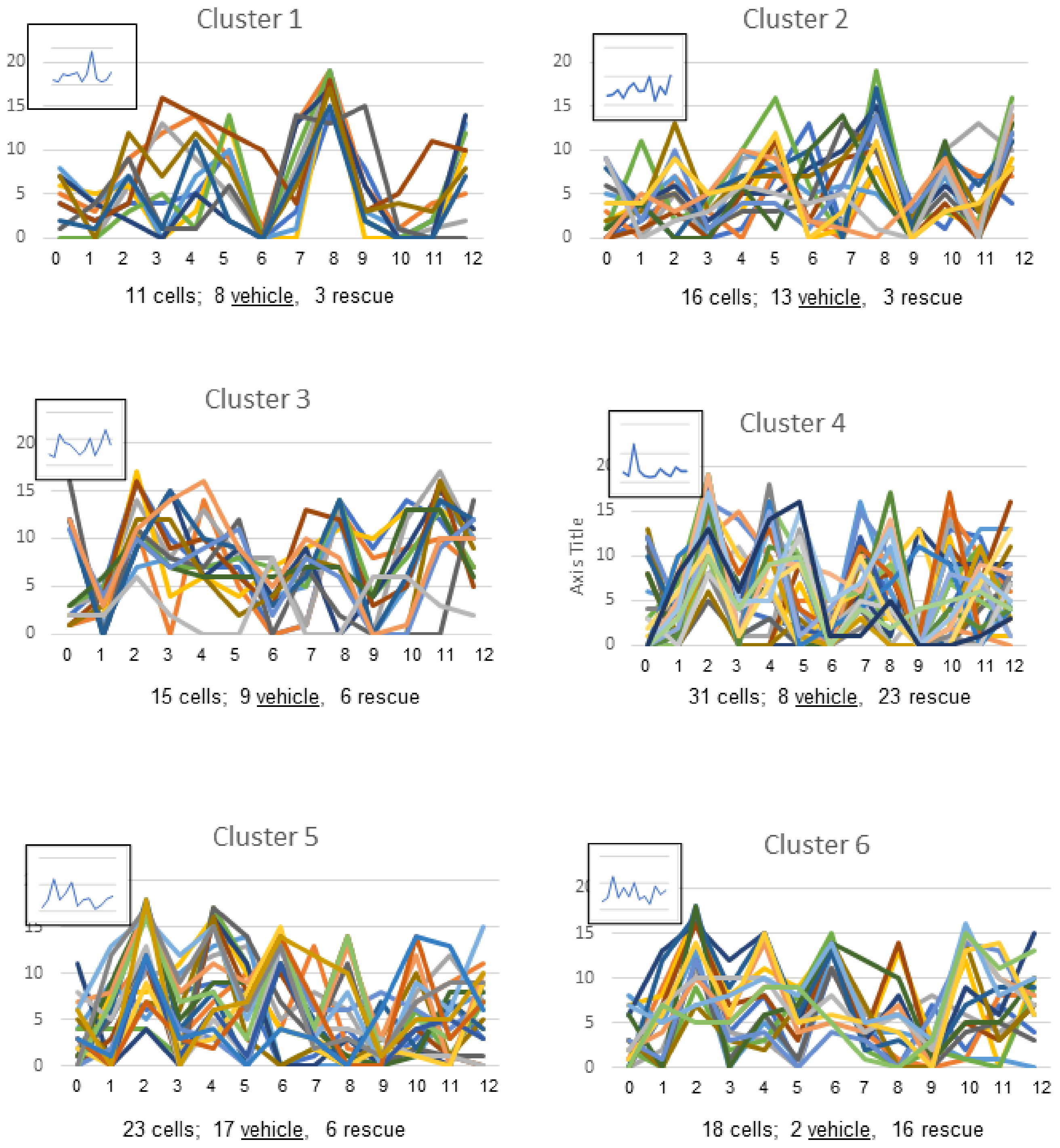

Graphs of cells in each male cluster. The CMP summary of all cells in a given cluster is shown from the 114 AM from male mice (29). The summary statistics indicating total cell numbers and number of vehicle- and SP-A treated cells in each cluster are given.

SUPPLEMENTARY TABLE S1

Supp. Table 1 (all males – 6 clusters – 13 markers)

Number of Vehicle and SP-A1 rescued cells/cluster (from Supp. Fig 4)							
Cluster	1	2	3	4	5	6	Total
Vehicle	8	13	9	8	17	2	60
Rescue	3	3	6	23	6	16	54
Total	11	16	15	31	23	18	114

SUPPLEMENTARY TABLE S1: Composition of Male AM cluster analysis.

The male AM data were subjected to cluster analysis and the composition (Vehicle or SP-A1 rescue) of each cluster is shown in this table. When all 114 AM from the male samples were subjected to cluster analysis based on their CMP content, 6 clusters were defined and the makeup of each cluster is shown in this supplementary table.
